# Contributions of Interleukin‐33 and TSLP in a papain‐soaked contact lens‐induced mouse conjunctival inflammation model

**DOI:** 10.1002/iid3.189

**Published:** 2017-07-20

**Authors:** Jobu Sugita, Yosuke Asada, Waka Ishida, Satoshi Iwamoto, Katsuko Sudo, Hajime Suto, Toru Matsunaga, Ken Fukuda, Atsuki Fukushima, Norihiko Yokoi, Tatsukuni Ohno, Miyuki Azuma, Nobuyuki Ebihara, Hirohisa Saito, Masato Kubo, Susumu Nakae, Akira Matsuda

**Affiliations:** ^1^ Laboratory of Ocular Atopic Diseases Department of Ophthalmology Juntendo University School of Medicine Tokyo Japan; ^2^ Japan Frontier Research Initiative, Institute of Medical Science University of Tokyo Tokyo Japan; ^3^ Department of Ophthalmology Kochi University School of Medicine Nankoku Japan; ^4^ Animal Research Center Tokyo Medical University Tokyo Japan; ^5^ Department of Dermatology Juntendo University School of Medicine Tokyo Japan; ^6^ Atopy Research Center Juntendo University School of Medicine Tokyo Japan; ^7^ SEED contact lens CO. Ltd. Tokyo Japan; ^8^ Department of Ophthalmology Kyoto Prefectural University of Medicine Kyoto Japan; ^9^ Department of Allergy and Immunology, Department of Molecular Immunology, Graduate School of Medical and Dental Science Tokyo Medical and Dental University Tokyo Japan; ^10^ National Research Institute for Child Health and Development Tokyo Japan; ^11^ Division of Molecular Pathology, Research Institute for Biological Sciences Tokyo University of Sciences Chiba Japan

**Keywords:** Basophil, IL‐33, ILC2, papain‐induced conjunctivitis, TSLP

## Abstract

**Introduction:**

Pathological changes of severe chronic allergic conjunctivitis are driven not only via acquired immunity but also via innate immunity. Type 2 immune response‐initiating cytokines may play some roles as innate immunity‐dependent components of the ocular surface inflammation. To investigate the involvement of type 2 immune response‐initiating cytokines in innate immunity‐dependent, papain‐induced conjunctival inflammation model using IL‐25‐, IL‐33‐, and TSLP receptor (TSLPR)‐knockout (KO) mice with reference to basophils and ILC2.

**Methods:**

Papain‐soaked contact lenses (papain‐CLs) were installed in the conjunctival sacs of C57BL/6‐IL‐25 KO, IL‐33 KO, TSLPR KO, Rag2 KO, Bas‐TRECK, and wild‐type mice and their eyes were sampled at day 5. The eosinophil and basophil infiltration in papain‐CL model was evaluated histologically and cytokine expression was examined. To clarify the roles of basophils and ILC2, basophil/ILC2‐depletion experiments were carried out.

**Results:**

Papain‐induced conjunctival inflammation exhibited eosinophil infiltration and upregulation of Th2 cytokine expression. Reduction of eosinophil and basophil infiltration and attenuated Th2 cytokine expression were observed in the papain‐CL model using IL‐33 KO and TSLPR KO mice. Depletion of basophils or ILC2s in the conjunctivae of the papain‐CL model reduced eosinophil infiltration.

**Conclusions:**

Innate immunity‐driven type 2 immune responses of the ocular surface are dependent on IL‐33, TSLP, basophils, and ILC2. These components may be possible therapeutic targets for refractory allergic keratoconjunctivitis.

## Introduction

Type 2 immune responses mediate inflammatory responses against various allergens (often with protease activity), parasites and large inert particulate structures causing tissue damage 1, 2. Interleukin‐33 (IL‐33), IL‐25, and thymic stromal lymphopoietin (TSLP) are regarded as type 2 immune response‐initiating cytokines 3. The cytokines are produced and released from epithelial cells in response to allergens, parasites, and tissue damage. Recently, we investigated the roles of type 2 immune response‐initiating cytokines in ragweed‐induced experimental allergic conjunctivitis (ragweed EAC) using IL‐33‐, IL‐25‐, and TSLP receptor (TSLPR)‐knockout (KO) mice 4. We found attenuated eosinophil infiltration and reduced Th2 cytokine (*il4, il5*, and *il13*) mRNA expression in the ragweed EAC model using IL‐33 KO mice, suggesting an indispensable role for IL‐33 in the pathophysiology of ragweed EAC mostly driven by adaptive immune responses 5.

On the other hand, pathological changes of severe chronic allergic conjunctivitis are driven not only via acquired immunity but also via innate immunity, because it was reported that mechanical damage (i.e., eye rubbing) 6, and ocular surface infection could exacerbate atopic keratoconjunctivitis (AKC) 7. Cases of AKC and vernal keratoconjunctivitis (VKC) refractory to glucocorticoid and immunosuppressive drug treatments have been reported 8. Such treatments mainly target adaptive immunity‐dependent components of conjunctival immune responses, so we considered that innate immunity‐dependent components of allergic conjunctivitis might be related to the pathophysiology of refractory AKC/VKC cases. We also reported upregulation of type 2 immune response‐initiating cytokines (IL‐33 and TSLP) expression in the tissues of giant papillae obtained from patients with refractory VKC and AKC 9, 10.

To clarify the roles of innate immunity‐dependent arms of type 2 immune responses on the ocular surface, we made a papain‐soaked soft contact lens (papain‐CL)‐ induced mouse conjunctivitival inflammation model. This model was based on our experiences with a papain‐induced lung inflammation model 11 that was used for analysis of innate immunity‐dependent components of type 2 inflammation in the airway. In addition to type 2 immune response‐initiating cytokines, the roles of innate immune cells, including basophils 12 and type 2 innate lymphoid cells (ILC2) 13, were also reported previously. According to previous studies, the proliferation and activation of basophils 14 and ILC2 15 are supported by IL‐33, and basophils induce type 2 immune reactions through IL‐4 production 16, whereas ILC2s induce type 2 immune reactions through production of IL‐5 and IL‐13 17. To clarify the roles of basophils and ILC2s as downstream effector cells of type 2‐initiating cytokines in the papain‐CL model, depletion experiments were also carried out in this study.

## Materials and Methods

### Genetically‐modified mice used in this study

C57BL/6N‐IL‐33 KO mice 11, C57BL/6N‐IL‐25 KO mice 18, and C57BL/6J‐TSLPR KO mice 19 were generated as previously reported. C57BL/6 wild‐type mice were purchased from Japan SLC (Shizuoka, Japan). C57BL/6‐Rag2 KO mice were obtained from Taconic (Petersburgh, NY). C57BL/6N‐IL‐4‐deficient G4 homozygote mice 20 and C57BL/6J‐Bas‐TRECK mice were generated as previously reported 21. All of the animal experiments were approved by the Animal Research Committee of Juntendo University (No. 280016) and conformed to the ARVO Statement on the Use of Animals in Ophthalmic and Vision Research (http://www.arvo.org/about_arvo/policies/statement_for_the_use_of_animals_in_ophthalmic_and_visual_research/).

### Papain‐contact‐lens‐induced conjunctivitis

Negatively‐charged soft contact lenses (CLs) 2 mm in diameter were soaked with 25 mg/ml papain (Wako, Tokyo, Japan) for 24 h (papain‐CLs; Supplementary Fig. S1). Phosphate‐buffered saline (PBS) was used to dissolve the papain powder. After intraperitoneal anesthesia using pentobarbital, a papain‐CL was installed in the conjunctival sac of the right eye (at day 0), and the eyeid were sutured with 8–0 nylon (Mani, Tochigi, Japan). Two days after the surgery (day 2), the papain‐CL was removed and another newly prepared papain‐CL was inserted in the same conjunctival‐sac and the eyelids were resutured. For negative control experiments, heat‐inactivated papain‐CLs or PBS‐soaked CL were used instead of papain‐CLs. Three days after the second surgery (day 5), the papain‐CL was removed and the eye and eyelid were enucleated and fixed using 4% paraformaldehyde‐PBS. Five IL‐33 KO, IL‐25 KO and TSLPR KO mice and five wild‐type mice were used for histological analysis. Another three IL‐33 KO mice, IL‐25 KO and TSLPR KO mice and wild‐type mice were used for real‐time PCR analysis. These experiments are repeated three times and the representative data was shown. Control experiments using Rag2 KO mice, and heat‐inactivated papain‐CL models were carried out separately using five mice for each group.

### Histological analysis

Eyes with the attached eyelids were fixed and embedded in paraffin and serial sections were made.

### Real‐time PCR analysis

Conjunctival tissue obtained from the papain‐CL model was immediately stored with RNA Later (Ambion, Austin, TX) to protect the RNA. Total RNA was extracted from the tissue with a NucleoSpin® II RNA isolation kit (Macherey‐Nagel GmbH, Duren, Germany). cDNAs were prepared using random primers and ReverTra Ace® reverse transcriptase (both from Toyobo, Osaka, Japan) according to the manufacturer's protocol. Real‐time PCR primers specific for mouse *il4*, *il5*, *il13*, *il33*, *tslp*, *ccl5* (encoding rantes), *ccl11*(encoding eotaxin), and *gapdh* genes were designed by QuantPrime (http://quantprime.mpimp-golm.mpg.de/), as shown in the Supplementary Table S1. Real‐time PCR analysis was performed with the ABI PRISM 7300 HT Sequence detection system using FAST‐SYBR green master mix (from Life Technology Japan, Tokyo, Japan). The relative gene expression was quantified by comparative Ct methods using *gapdh* expression in the same cDNA as the controls.

### Immunohistochemistry

Immunohistochemical analysis was carried out as previously described 4. In brief, for detection of basophils, a rat anti‐mMCP8 antibody (BioLegends, San Diego, CA) was used as the primary antibody and a donkey‐Alexa 488‐conjugated anti‐rat IgG antibody (from Life Technologies) was used as the secondary antibody.

### ILC2 detection by flow cytometric analysis

Cervical lymph node, lacrimal gland, and conjunctival tissues obtained from the papain‐CL model in the wild‐type mice were subjected to flow cytometric analysis (FACS). Mouse lacrimal glands (LG) and conjunctival tissues (*n* = 10) obtained from papain‐CL model mice were dissected using microscissors under a stereomicroscope. The tissues were minced into small fragments, followed by digestion with 1 mg/ml collagenase type IV and 0.5 mg/ml DNase I (Roche, Basel, Switzerland) for 15 min at 37°C. The tissue suspension was triturated using a syringe and filtered through a 70 μm cell strainer (BD Biosciences, San Jose, CA). Cells were thoroughly washed and resuspended in Hank's balanced salt solution containing 2% fetal bovine serum (2% FBS in HBSS, both from Life Technology). Cells were reacted with an anti‐Fc receptor blocking reagent and washed before staining. Then mouse LG and conjunctival tissues were stained with Brilliant Biolet 421‐anti‐mouse‐CD45(30‐F11), PE‐conjugated anti‐mouse lineage marker mAbs (CD3[145‐2C11], CD19[6D5], NK1.1[PK136], CD11b[M1/70], Gr1[RB‐8C5], and Ter119[Ter‐119]), FITC‐conjugated anti‐mouse ST2L ([DJ8], MD Bioscience, Zurich, Switzerland), Cy7‐conjugated anti‐mouse CD127(A7R34), APC‐conjugated anti‐mouse CD25(PC61), and APC‐Cy7‐conjugated anti‐mouse CD90.1(30‐H12). All the conjugated Abs were purchased from BioLegends unless otherwise stated. All the samples were analyzed using MAQS Quant® (Milteny Biotech, Germany).

### Basophil‐depletion experiments

For basophil depletion in vivo 21, Bas‐TRECK transgenic mice were injected with 250 ng of intraperitoneal diphtheria toxin (DT) injection at day‐1 and day 2. To evaluate the effect of basophil depletion in the papain‐CL model, five Bas‐TRECK mice were treated with DT, another five were used without DT treatment, and five C57BL/6 wild‐type mice were used as a positive control. Three additional mice for each group were used for cytokine expression analysis.

### ILC2‐depletion experiments

For depletion of ILC2s in vivo 17, Rag2 KO mice were intraperitoneally injected with 200 µg of rat anti‐CD25 mAb (clone PC61) (ten mice) and a rat IgG (Jackson ImmunoResearch Laboratories, Inc.,) as controls (ten other mice) 2 days before papain‐CL insertion.

### Statistical analysis

Statistical evaluations were performed with the two‐tail unpaired Mann–Whitney *U* test. *P *< 0.05 was considered statistically significant.

## Results

Papain soaked soft contact lens (papain‐CLs, Supplementary Fig. S1) were installed in the right conjunctival sacs of mice (Supplementary Fig. S2A), and the eyelids were sutured with 8–0 nylon on day 0 (Supplementary Fig. S2B). After the CL removal and reinsertion on day 2, the second papain‐CL was removed on day 5. Lid swelling was observed in the papain‐CL model using wild‐type mice (Supplementary Fig. S2C). We then counted the number of the infiltrating eosinophils using Giemsa staining sections (Supplementary Fig. S3). There was no significant difference in the number of infiltrating eosinophils between wild‐type mice and Rag2 KO mice (Supplementary Fig. S4A, B, and D). In separated series of experiments, we observed reduction of the number of infiltrating eosinophils in the wild‐type mice conjunctiva treated by heat‐inactivated papain‐CL compared to those treated by the intact papain‐CL (Supplementary Fig. S4C and E), and further reduction of the number of infiltrating eosinophils in the wild‐type mice conjunctiva by lid suture only (data not shown). There was no significant upregulation of the total IgE concentration after papain‐CL administration (Supplementary Fig. S5).

**Figure 1:**
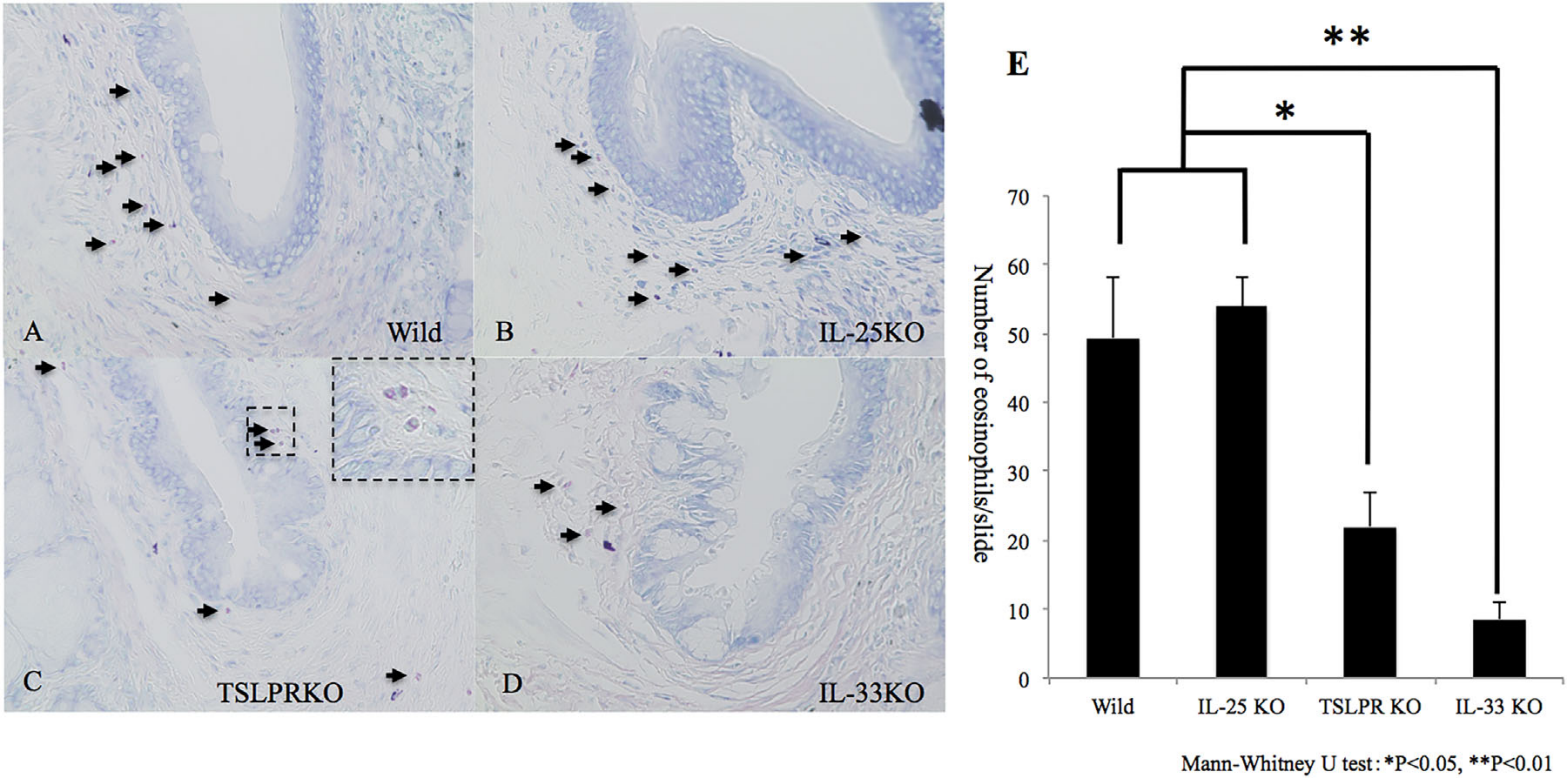
The infiltrating eosinophils were counted by Giemsa staining. Significant reduction of the eosinophil numbers was observed in the models using TSLPR KO (C) and IL‐33 KO mice (D) compared to wild‐type (A) and IL‐25 KO (B) mice. The infiltrating eosinophils were shown at higher magnification in the inset (dotted box in C). Numbers of eosinophils in the conjunctivae per slide are shown (E). Error bars show means ± SD (*P < 0.05, *n* = 5 per group). Representative data from three independent experiments are shown.

**Figure 2:**
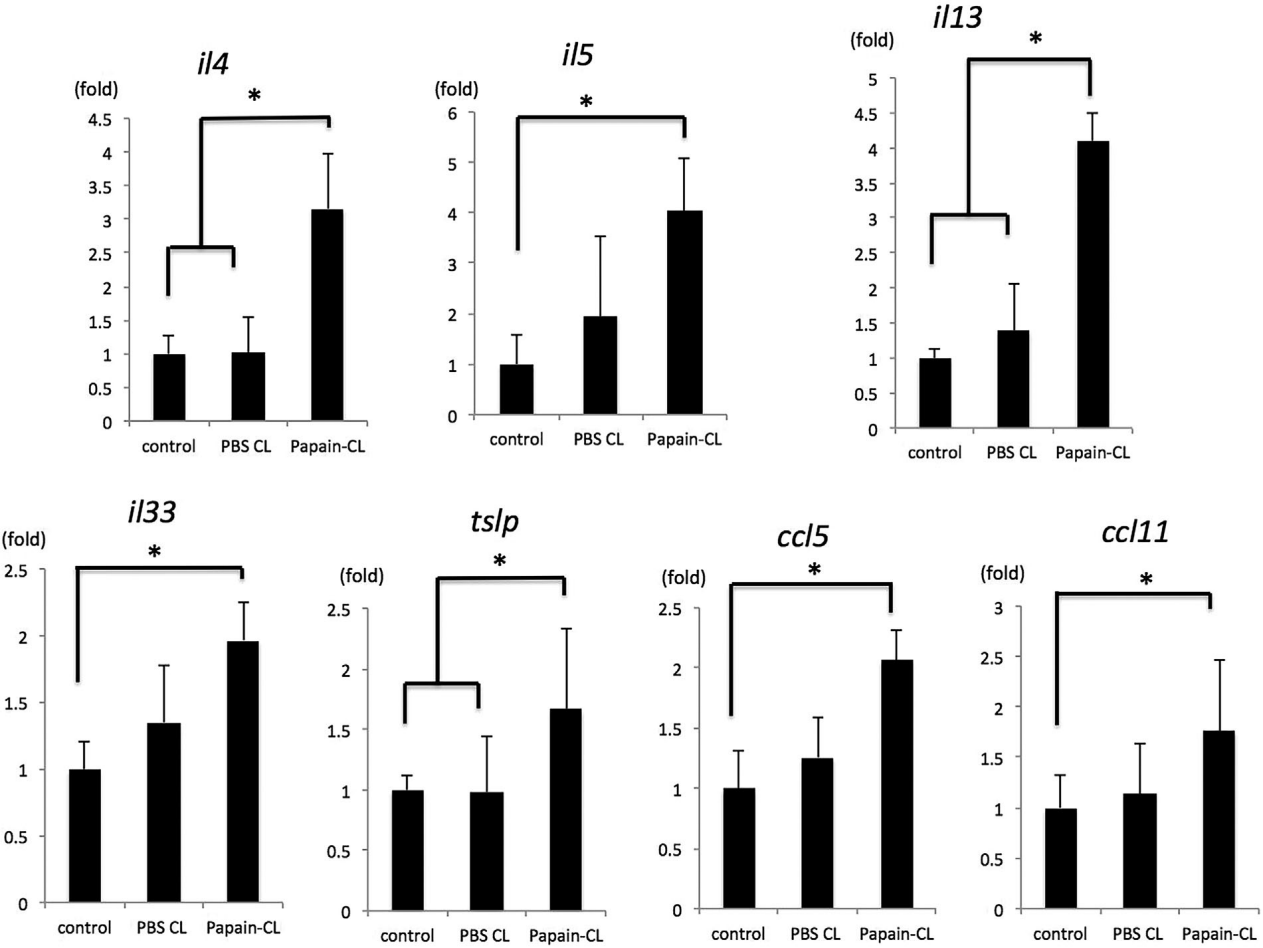
Expression of inflammatory cytokine/chemokine mRNAs in papain‐CL model using wild‐type mice was quantified by real‐time PCR. Relative mRNA expression is shown as fold changes of mRNA expression levels of control conjunctival tissue. Significantly elevated expression of *il4, il5, il13*, *il33, tslp, ccl5,* and *ccl11* mRNAs was observed in the papain‐CL‐inserted conjunctiva. Error bars show means ± SD. (*P < 0.05, n = 3 per group). Representative data from three independent experiments are shown.

Significantly reduced numbers of infiltrating eosinophils were observed in IL‐33 KO mice and TSLPR KO mice compared to wild‐type mice during in papain‐CL model (Fig. 1A,C–E). There was no significant difference in the numbers of infiltrating eosinophils between IL‐25 KO mice and wild‐type mice (Fig. 1B and E). Significant upregulation of *il4*, *il5*, *il13*, *il*33, *tslp*, and *ccl5, ccl11* mRNA expression (Fig. 2) was observed in the conjunctival tissue obtained from the papain‐induced inflammation model using wild‐type mice compared to the contralateral conjunctival tissue of wild‐type mice (control). Significant upregulation of *il4*, *il13*, and *tslp* expression in papain‐CL‐challenged conjunctival tissue was also noted as opposed to mock (PBS‐CL)‐challenged conjunctivae. On the other hand, expression of *il5, il33, ccl5,* and *ccl11* was significantly upregulated only compared to the control conjunctivae. In addition, significant attenuation of *il4*, *il5*, and *il13* mRNAs was observed in the papain‐CL models using IL‐33 KO compared to the model using wild‐type mice (Supplementary Fig. S6). In case of TSLPR KO mice (Supplementary Fig. S7), significant attenuation of *il4* and *il5* mRNAs was observed in the papain‐CL models compared to the model using wild‐type mice.

**Figure 3:**
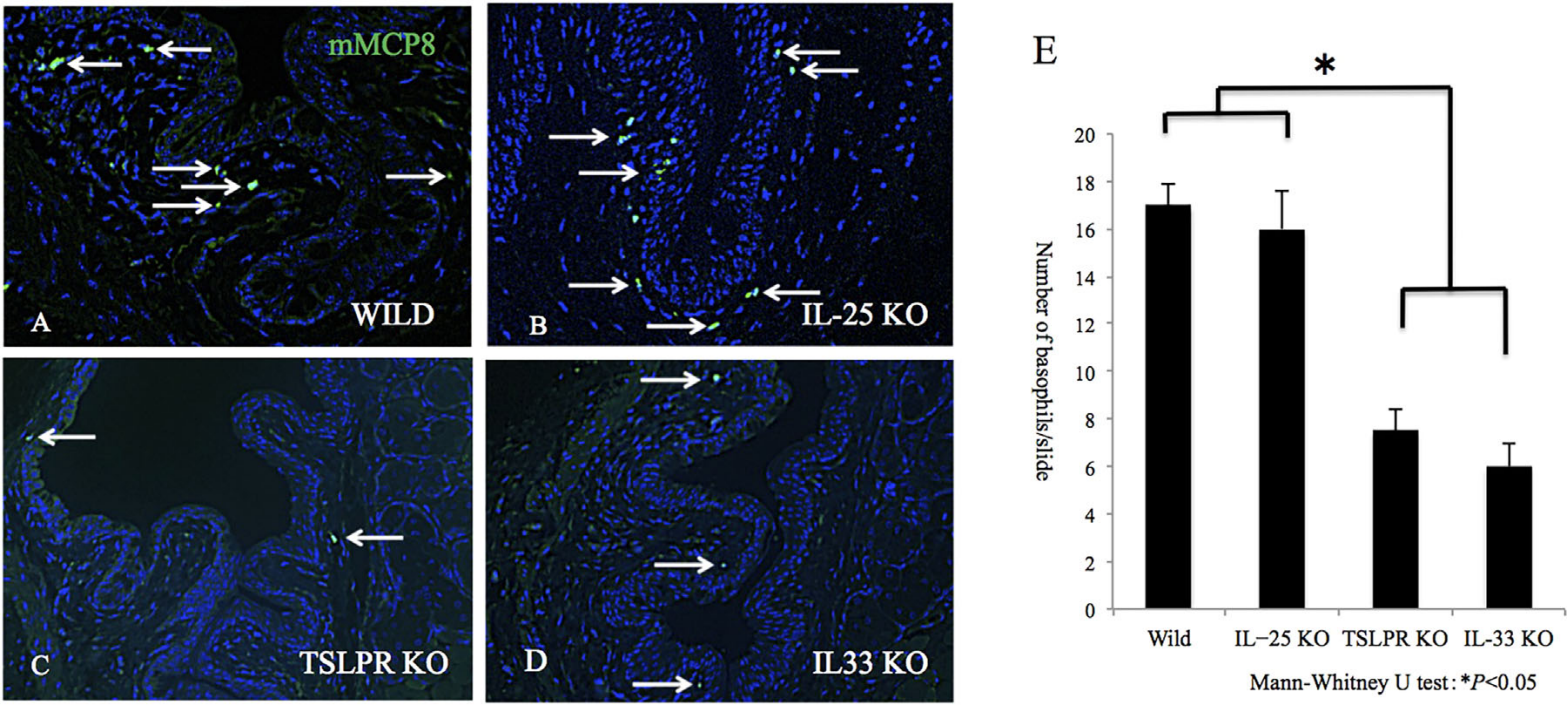
Reduced numbers of mMCP8‐positive basophils (arrows) were found in the papain‐CL models using TSLPR KO (C) and IL‐33 KO mice (D) compared to wild‐type (A) and IL‐25 KO mice (B). Original magnification x200. Numbers of the basophils in the conjunctivae per slide are shown (E) (*P < 0.05, *n* = 5 per group). Error bars show means ± SD. Representative data from three independent experiments are shown.

**Figure 4:**
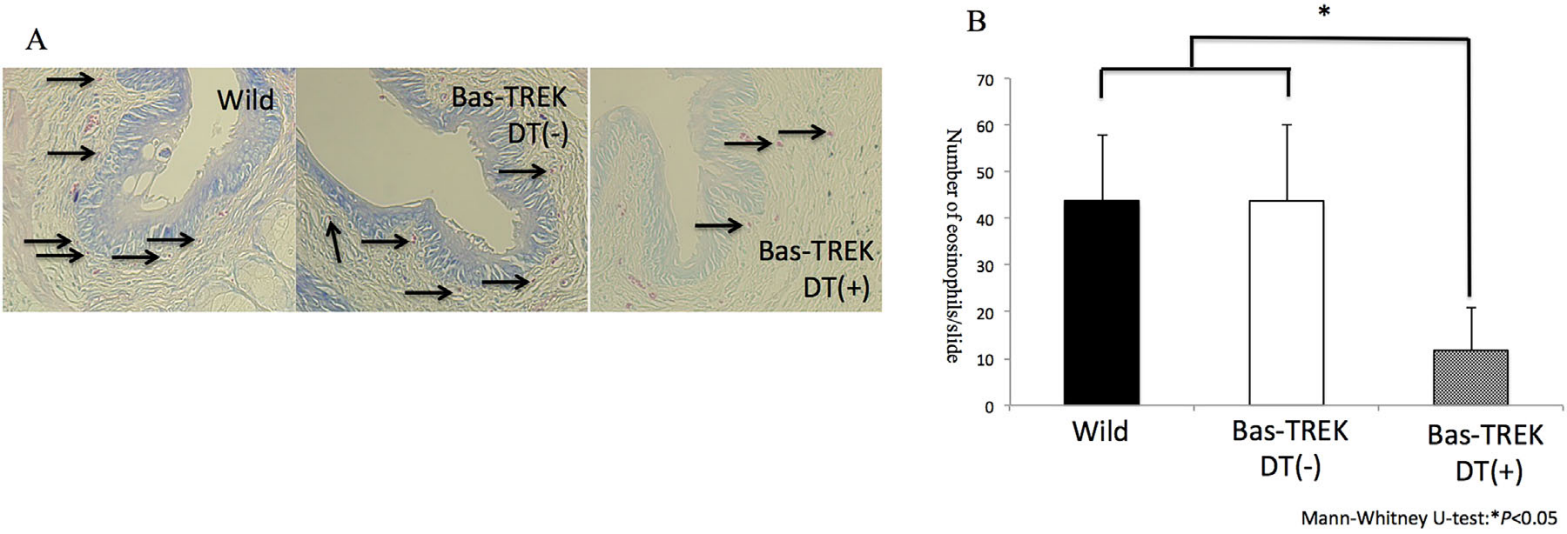
Basophil depletion reduced eosinophil infiltration in papain‐CL model. Diphtheria toxin (DT) injection at day—1 and day 2 resulted in depletion of basophils in the conjunctiva (A). The eosinophils in papain‐CL model using Bas‐TRECK mice were significantly diminished by DT treatment compared to those in mice without DT treatment and wild‐type mice. (B) (*P < 0.05, n = 5 per group). Error bars show means ± SD. Representative data from two independent experiments are shown.

Anti‐mMCP8 immunohistochemical staining was performed to quantify the numbers of infiltrating basophils in papain‐CL‐induced inflammation model. Significantly reduced basophil infiltration (Fig. 3E) was observed in the conjunctival tissues of TSLPR KO (Fig. 3D) and IL‐33 KO mice (Fig. 3C) compared to those of the wild‐type mice. There was no significant difference between IL‐25 KO mice and wild type mice (Fig. 3B). Using anti‐mMCP8 immunohistochemical staining, we confirmed depletion of basophils in the conjunctival tissue by intraperitoneal injection of DT in Bas‐TRECK mice (Supplementary Fig. S8). The numbers of infiltrating eosinophils in papain‐CL models were significantly diminished by the basophil depletion using DT administration compared to non‐treated control Bas‐TRECK mice (Fig. 4A and B). Significant reduction of *il4, il5, il13, and ccl11* mRNA expression was observed in the papain‐CL model using Bas‐TRECK mice with DT treatment compared to those without DT treatment (Supplementary Fig. S9). Reduction of infiltrating eosinophil numbers was also observed in papain‐CL model in IL‐4‐deficient mice (Supplementary Fig. S10A).

**Figure 5:**
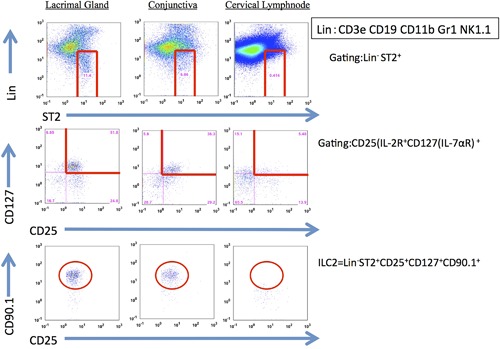
Detection of ILC2 in the mouse conjunctival tissue by flow cytometry. ILC2s (Lineage^−^ST2L^+^CD25^+^CD127^+^CD90.1^+^) were identified in the conjunctival tissue (bottom left) and lacrimal gland tissue (bottom center) from papain‐CL model. On the other hand, only a few ILC2s were detected in the cervical lymph node (bottom right). Representative data from two independent experiments are shown.

**Figure 6:**
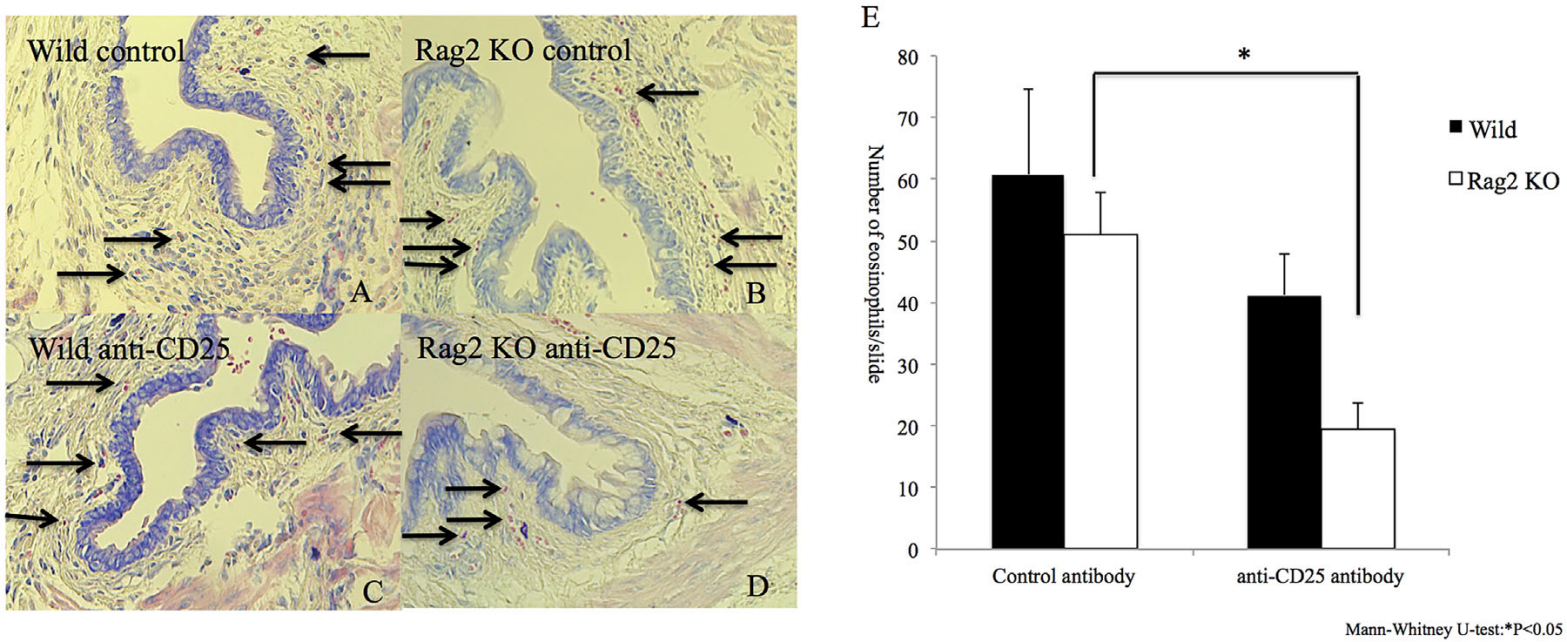
Attenuated eosinophil infiltration in the papain‐CL model was observed after ILC2 depletion. The numbers of eosinophils in papain‐CL model were counted by Giemsa staining (A, B, C, D). The number of infiltrating eosinophils in Rag 2 KO mice (E) was significantly diminished by the anti‐CD25 mAb treatment (*P < 0.05, *n* = 5 per group). Error bars show means ± SD. Representative data from two independent experiments are shown.

We detected ILC2s (lineage^−^ST2^+^CD25^+^CD127^+^CD90.1^+^) in the conjunctival tissues and lacrimal gland tissues from mice with papain‐CL model (Fig. 5, lower lane). On the other hand, only a few ILC2s were detected in the cervical lymph nodes of the same model. Intraperitoneal injection of the anti‐CD25 mAb resulted in depletion of ILC2s in the conjunctival tissue of Rag2 KO mice (Supplementary Fig. S11). The numbers of infiltrating eosinophils in papain‐CL models were significantly diminished by ILC2 depletion using the anti‐CD25 antibody compared to isotype‐matched control antibody administration (Fig. 6).

## Discussion

In this study, we made a conjunctival inflammation model using papain‐CLs (Supplementary Figs. S1 and 2). This conjunctival inflammation model is mostly dependent on innate immune systems because a comparative degree of eosinophil infiltration was observed in this model using Rag2 KO mice (Supplementary Fig. S4), which lack acquired immune systems 22. To develop a papain‐CL‐induced conjunctival inflammation model, we performed preliminary experiments and found that only negatively charged‐CL could retain papain (Supplementary Fig. S1), because the papain molecule (pI = 8.75) has a positive charge in neutral pH (pH7.0). Based on this result, we used a negatively charged‐CL, trimmed by trepaning to 2 mm in diameter to fit the conjunctival sac of the mouse eye, and used an 8–0 nylon suture to prevent dislocation of the papain‐CL (Supplementary Fig. S2B). To make the degree of inflammation reproducible and prevent papain‐CL dislocation, we changed the papain‐CL once on day 2 and analyzed the eye on day 5. We counted the number of eosinophils from the corneal limbus to the mucocutaneous junction via the conjunctival fornix, taking special care to avoid the effect of suture‐induced inflammatory cell infiltration (Supplementary Fig. S3). We found attenuated eosinophil infiltration in the models using TSLPR KO and IL‐33KO mice compared to that with wild‐type mice (Fig. 1E). Our results showed the indispensable roles of IL‐33 and TSLP in the eosinophil infiltration induced by papain‐CL, this result is consistent with previous reports showing attenuated papain‐induced allergic airway inflammation in IL‐33 KO mice 11, and reduced papain‐induced allergic inflammation in the lung of TSLPR KO mice 23. In the case of ragweed induced experimental allergic conjunctivitis, we evaluated clinical scores (lid edema, conjunctival hyperemia, and swelling) as parameters to grade severity of inflammation 4. However, in the case of papain‐CL model, it was difficult to obtain reliable clinical scores because the inflammation of this model did not highly depend on immediate hypersensitivity reactions. As in the case of papain‐induced lung inflammation analaysis 11, 16, 17, 24, in which the clinical parameters for asthma were lacking, the papain‐CL model could not be evaluated using clinical scores. Nevertheless, via enumeration of inflammatory cells and by quantification of cytokine expression, the papain‐CL model seems to be useful to evaluate innate immunity‐dependent components of type 2 inflammation. We quantified the serum total IgE concentrations before and after papain‐CL challenges, and found no significant IgE increase for any of the mouse types used in this study (Supplementary Fig. S5). This result was in agreement with a previous report showing no apparent increase of serum IgE at 6 days after intranasal administration of 10 µg of papain compared to heat‐inactivated control papain administration 24. These results suggested that the 5‐day‐long papain‐CL model was not dependent on acquired immunity or on a systemic increase of IgE.

Real‐time PCR analysis revealed that *il33* mRNA expression was significantly upregulated in the papain‐CL model (Fig. 2) and there was a moderate degree of *il33* mRNA upregulation induced by PBS‐soaked CL insertion. These results suggested that mechanical damage induced by CL insertion itself was partially responsible for the *il33* mRNA induction in the papain‐CL model, consistent with a previous report, showing the roles of IL‐33 as an “alarmin,” in which mechanical injury induced IL‐33 expression 25. Attenuated Th2 cytokines (*il4, il5,* and *il13*) mRNA expression was observed in the papain‐CL model using IL‐33 KO (Supplementary Fig. S6) and TSLPR KO (Supplementary Fig. S7) mice but not for IL‐25 KO mice (data not shown). These results were in agreement with the indispensable roles of IL‐33 and TSLP in the induction of eosinophilc infiltration in the papain‐CL model (Fig. 1).

Among the IL‐33 or TSLP‐responding myeloid cells (i.e.*,* eosinophils, basophils, mast cells, and macrophages) 26, we investigated the roles of basophils in papain‐CL model because they are known to produce a large amount of IL‐4 12. We confirmed infiltration of mcp8‐positive basophils in the papain‐CL model (Fig. 3A) and reduction of basophils in IL‐33 KO (Fig. 3C) and TSLPR KO (Fig. 3D) mice. The reduced numbers of basophils in IL‐33 KO and TSLPR KO mice might be related to an attenuated IL‐4 expression in the papain‐CL model (Supplementary Figs. S6 and 7), and reduced type 2 immune responses in papain‐CL model in these mice. Furthermore, we showed depletion of basophils (Supplementary Fig. S8) was accomplished by DT administration in Bas‐TRECK mice, and eosinophil infiltration was reduced in the papain‐CL conjunctivitis using DT‐treated Bas‐TRECK mice (Fig. 4A and B). In addition, reduced eosinophil infiltration was observed in IL‐4 KO (G4) mice 20 compared to wild‐type mice (Supplementary Fig. S10). Taken together, our results suggested that IL‐33 and TSLP signaling pathway are indispensable for papain‐CL induced type 2 inflammation through IL‐4 signaling and basophil infiltration.

These roles of basophils for innate immunity‐dependent ocular surface type 2 inflammation were further supported by our previous reports showing basophil infiltration in AKC/VKC tissue 27.

The IL‐33‐dependent elevation of *il5* and *il13* mRNA expression in the papain‐CL model (Supplementary Fig. S6) prompted us to investigate the possible existence of type 2 innate lymphoid cells (ILC2s) in the conjunctival tissue of papain‐CL model because ILC2s are known to produce large amounts of IL‐5 and IL‐13 13, and also because the existence of IL‐33 is essential for ILC2 activation 28. Indeed, we confirmed the existence of ILC2s in mouse conjunctival tissues by FACS analysis (Fig. 5), suggesting that they could be one of the sources of upregulated *il5* and *il13* mRNA expression in papain‐CL model (Fig. 2). Interestingly, we also found ILC2s in the tissue of the extraorbital lacrimal gland (Fig. 5), the organ connected with the conjunctiva via ducts. We found a few ILC2s in the cervical lymph nodes, which is consistent with the results showing only a few ILC2s in the lymph nodes of papain‐stimulated lung tissue 17. To further clarify the role of ILC2s in the papain‐CL model, we carried out ILC2‐depletion experiments via intraperitoneal anti‐CD25 antibody administration, using Rag 2 KO mice as previously described 17. We found that more than 80% of ILC2s in the conjunctival tissue were depleted by the treatment (Supplementary Fig. S11), and significant reduction of the eosinophil infiltration in the conjunctivae of the papain‐CL model caused by ILC2 depletion (Fig. 6E). These results supported the indispensable role of ILC2s in the eosinophil infiltration in papain‐CL model. It also should be noted that decrease of eosinophil infiltration in Rag2 KO mice compared to wild type mice was observed under the influence of CD25 antibody (Fig. 6), suggesting at least some part of eosinophil infiltration in this model could be affected by cells of the adaptive immune system.

Our cell depletion experiments showed that basophil and ILC2 contributed to the eosinophil infiltration in the papain‐CL model. Although it is unknown how basophils and ILC2s interact in papain‐CL model, it may be possible that basophil‐derived IL‐4 induces *ccl11* expression in ILC2 and thereby increases eosinophil recruitment in the conjunctiva. The reduction of *ccl11* expression was demonstrated in a papain‐induced airway inflammation model using *il4* 3′UTR‐deficient mice (impaired IL‐4 expression specifically in basophils) by Motomura et al. 16. Consistent with this hypothesis, we found that the papain‐CL model using basophil‐depleted mice showed not only diminished *il4* mRNA expression (due to depletion of basophils) but also diminished *il5*, *il13,* and *ccl11* mRNA expression (Supplementary Fig. S9), suggesting that basophil depletion might attenuate activation of ILC2s, which are known to produce IL‐5, IL‐13, and eotaxin (encoded by *ccl11*).

In summary, we clarified that innate immunity‐dependent type 2 immune reactions played significant roles in conjunctival eosinophil infiltration via the IL‐33 and TSLP signaling pathways. Reduction of infiltrating basophils in the papain‐CL model using IL‐33 KO and TSLPR KO mice and the results of basophil‐depletion experiments suggested essential roles of IL‐33/TSLP‐activated basophils in papain‐CL‐induced conjunctival inflammation. We also showed ILC2s in the conjunctiva had an essential role in the papain‐CL‐induced type 2 immune reactions on the ocular surface. Limitation of our study is the relatively acute nature of the papain‐CL model, which lacks proliferative changes of conjunctival tissue as observed in severe chronic human allergic conjunctivitis. Nevertheless, we consider that our model is useful to further evaluate innate‐immunity‐dependent type‐2 inflammation of the ocular surface, which may be related to allergic inflammation of the ocular surface and lead to finding possible therapeutic targets for refractory cases.

## Conflicts of Interest

The authors do not have conflicts of interest for the content of this manuscript.

## Supporting information

Additional supporting information may be found in the online version of this article at the publisher's web‐site.

**Figure S1**. To evaluate the effect of the electrical charges of soft contact lenses (CLs) on the ability to retain papain, CLs (both negatively charged and positively charged) were incubated with papain solution for 24 h. Then the papain‐CLs were incubated with SDS sample buffer. Immunoblot analysis with an anti‐papain Ab showed that papain was retained on negatively‐charged CLs (left lane) even after PBS washing (middle lane), but not on positively‐charged CLs (right lane).
**Figure S2**. Papain‐contact lens (papain‐CL)‐induced inflammation in the mouse eye. On day 0, a papain‐CL was inserted into the conjunctival‐sac of the right eye (A), and the eyelid was sutured with 8‐0 nylon (B). The papain‐CL was exchanged once on day 2, and the second papain‐CL was removed and the eye was sampled for further analysis on day 5 (C).
**Figure S3**. We counted the numbers of eosinophil in the conjuctival epithelium and substantia propria, except within the lumina of vessels, through the corneal limbus to the mucocutaneous junction via the conjunctival fornix.
**Figure S4**. Giemsa staining showed comparable numbers of eosinophils (arrows) in wild‐type mice (A) and Rag2 KO mice (B), but significantly fewer infiltrating eosinophils were observed in wild‐type mice wearing heat‐inactivated papain‐CLs (C). Numbers of eosinophils in the conjunctivae per slide are shown (D). N.S.: No significant difference. Error bars show means ± SD (*P < 0.05, *n* = 5 per group) Representative data from two independent experiments are shown.
**Figure S5**. Total serum IgE levels on day 0 (before administration) and day 5 (after final papain‐CL removal) determined using ELISA MAX mouse IgE ELISA kits (Biolegend, San Diego, CA). When mice in the same genotype group were compered, there was no significant upregulation of the total IgE concentration after papain‐CL administration. The data are representative of triplicate measurements. Error bars show means ± SD (*P < 0.05, *n *= 3 per group). Representative data from three independent experiments are shown.
**Figure S6**. Cytokine/chemokine (il4, il5, il13, ccl5, ccl11, tslp) mRNA expression in the conjunctivae of papain‐CL models using wild‐type and IL‐33 KO mice was quantified by real‐time PCR. Relative mRNA expression is shown as fold changes of mRNA expression levels in contralateral conjunctival tissue (control) of the wild‐type mice. Data representative of triplicate measurements of three mice per group, carried out as three independent experiments, are shown. (*P < 0.05, **P < 0.01).
**Figure S7**. Cytokine/chemokine (il4, il5, il13, ccl5, ccl11, il33) mRNA expression in the conjunctivae of papain‐CL conjunctivitis models using wild‐type and TSLPR KO mice was quantified by real‐time PCR. Relative mRNA expression is shown as fold changes of mRNA expression levels in contralateral conjunctival tissue (control) of the wild‐type mice. Data representative of triplicate measurements of three mice per group, carried out as three independent experiments, are shown. (*P < 0.05).
**Figure S8**. mMCP8 immunostaining was carried out to confirm the efficiency of diphtheria toxin (DT) treatment‐induced basophil depletion in Bas‐TRECK mice. No basophils were observed in the papain‐CL model with DT treatment (B). On the other hand, basophils were detected in the papain‐CL model without DT treatment (A). Representative data from two independent experiments are shown. Original magnification ×200.
**Figure S9**. The expression of cytokines/chemokines (il4, il5, il13, tslp, il33, ccl5, and ccl11) in papain‐CL model was compared among wild‐type, Bas‐TRECK mice without DT treatment, and DT‐treated Bas‐TRECK mice. Significant reduction of il4, il5, il13, and ccl11 mRNA expression was observed in papain‐CL model using Bas‐TRECK mice. (*P < 0.05 and **P < 0.01, *n *= 3 per group). Representative data from two independent experiments are shown.
**Figure S10**. Reduction of infiltrating eosinophil numbers (A) was observed in the papain‐CL model in IL‐4 KO (G4) mice (*P < 0.05, *n *= 5 per group). Expression of inflammatory cytokine/chemokine (il4, il5, il13, ccl5, and ccl11) mRNAs was quantified by real‐time PCR. Significantly attenuated expression of il4 and il13 mRNAs was observed in the papain‐CL model IL‐4 KO mice compared to the wild‐type mice. No differential il5 and ccl5 mRNA expression was observed. Data representative of triplicate measurements, carried out as two independent experiments, are shown. (*P < 0.05, *n *= 3 per group).
**Figure S11**. Conjunctival tissues isolated from 10 eyes of isotype‐matched mAb‐treated Rag2 KO mice and from 10 eyes of anti‐CD25 mAb‐treated Rag2 KO mice were analyzed by FACS (A). Total numbers of ILC2s detected in the conjunctival tissues were counted (B). Representative data from two independent experiments are shown.
**Table S1**. Primer pairs used in this study.
