# Supplementary material for: Contributions of Interleukin‐33 and TSLP in a papain‐soaked contact lens‐induced mouse conjunctival inflammation model
Source: Immun Inflamm Dis. 2017 Jul 20;5(4):515–25. doi: 10.1002/iid3.189 (PMC5691312; doi:10.1002/iid3.189)

**Supplementary Figure 1: Western blot analysis showing papain retention in**

**papain-soaked negatively charged contact lenses**

**
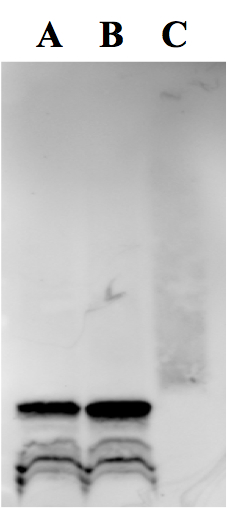
**

**Supplementary Figure 2: Papain CL-induced inflammation**

**
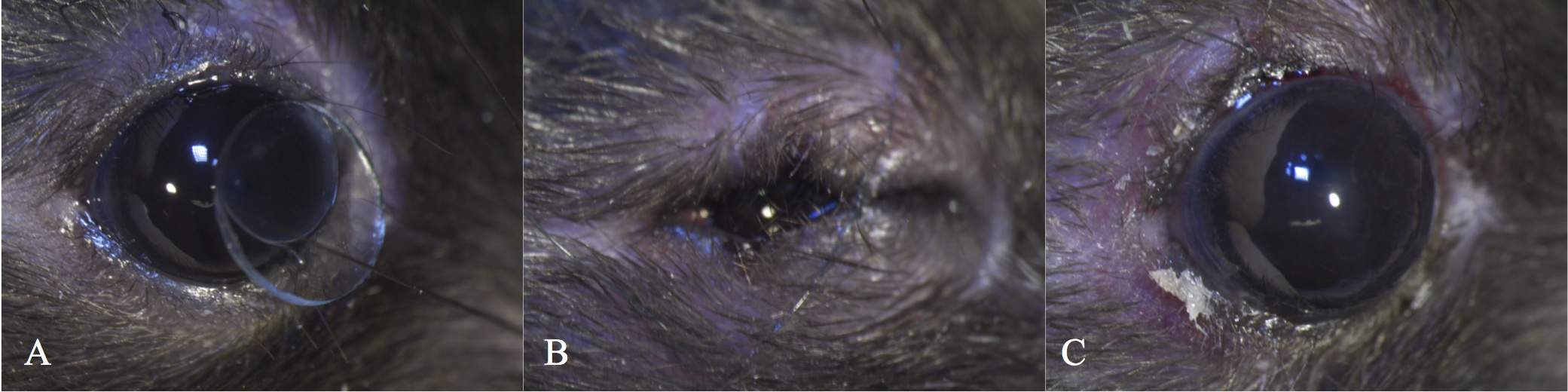
**

**Supplementary Figure 3: Eosinophil counting method used in this model**


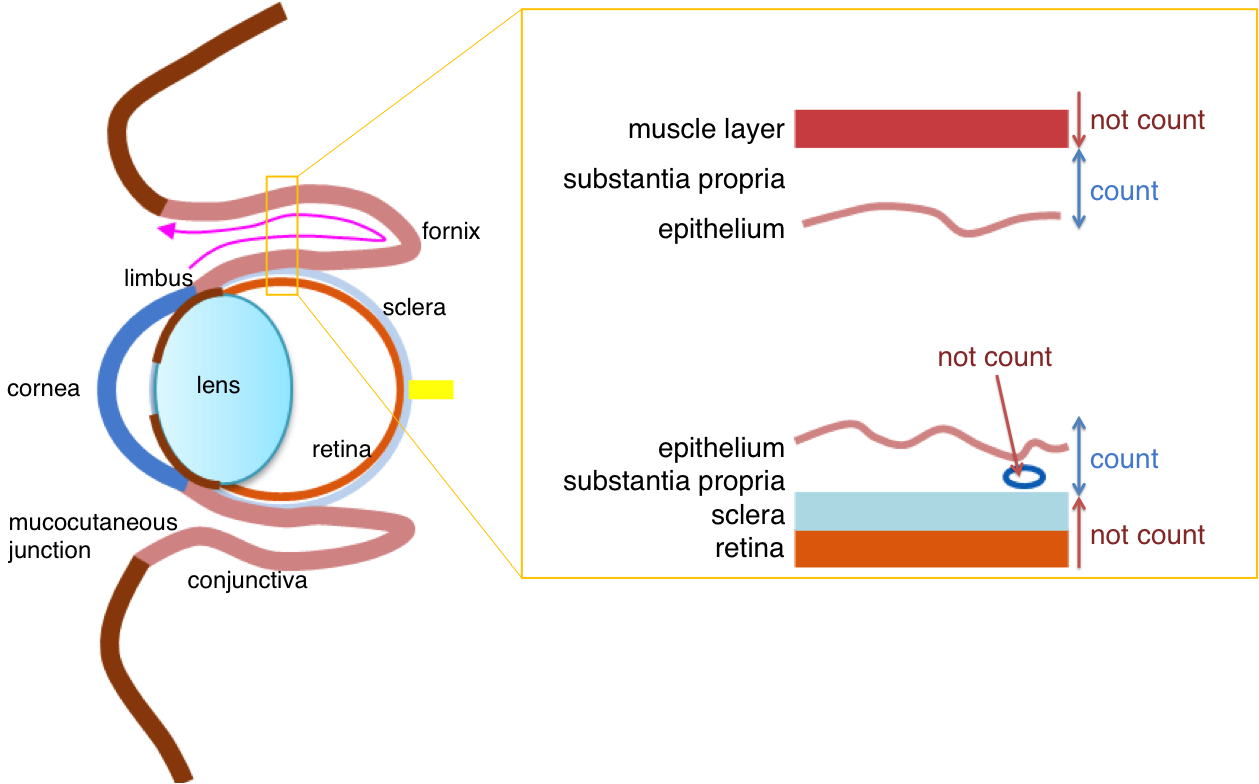


**Supplementary Figure 4: Eosinophil infiltration in papain-CL model using Rag2 KO mice shows comparable numbers of infiltrating eosinophils**

**
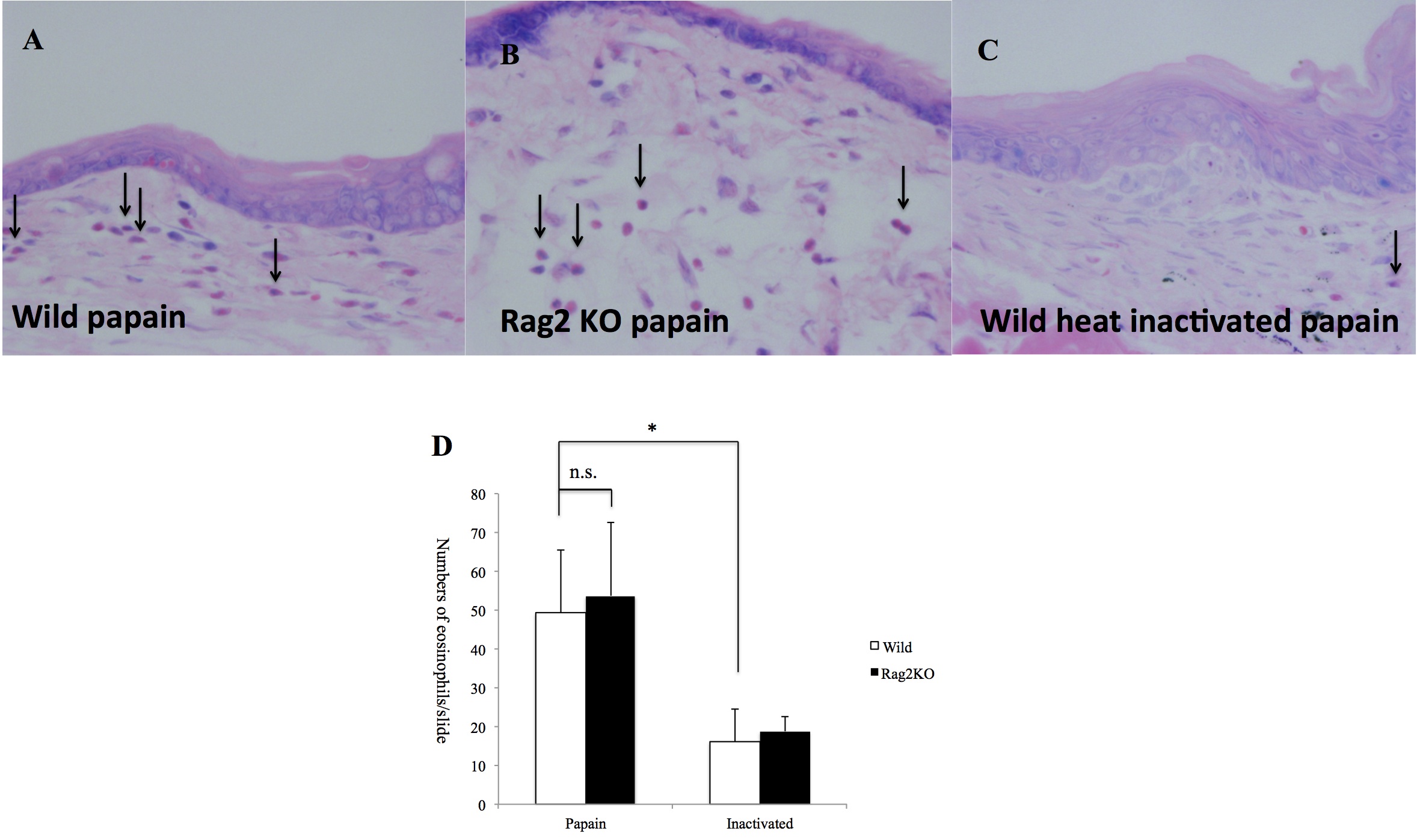
**

**Supplementary Figure 5: Quantification of total serum IgE before and after 5-day papain-CL challenges**

**
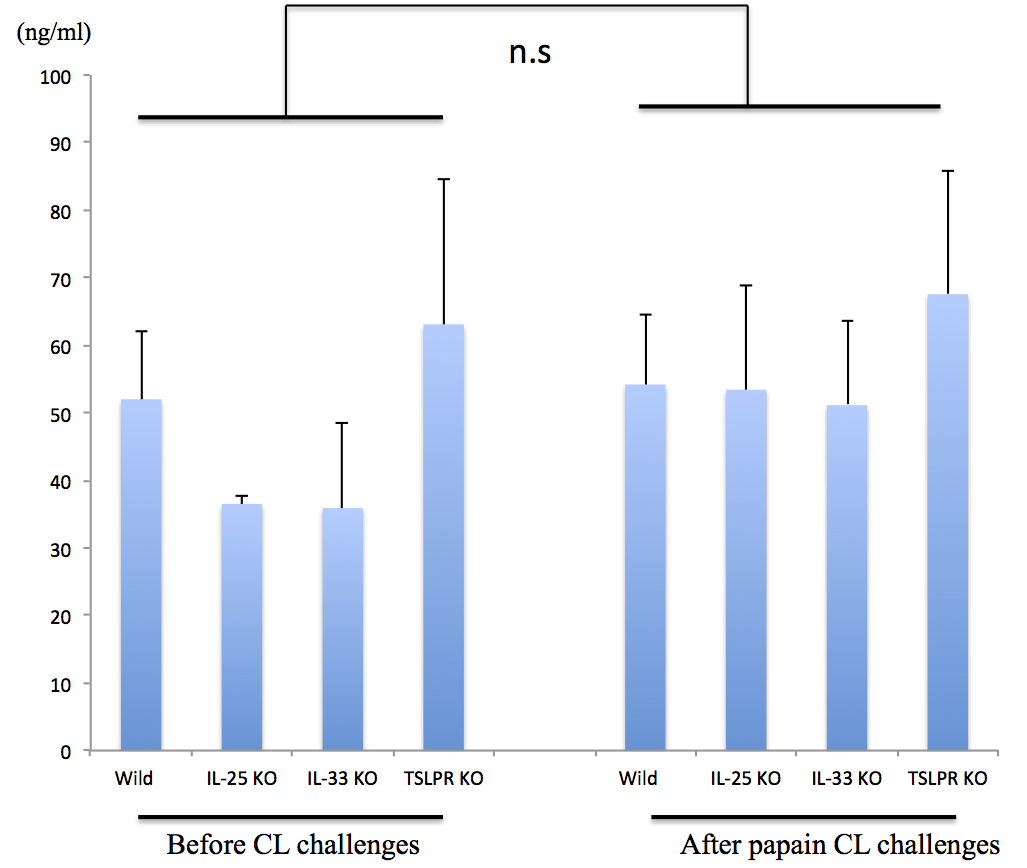
**

**Supplementary Figure 6: Cytokine expression in papain-CL model using IL-33 KO mice**

**
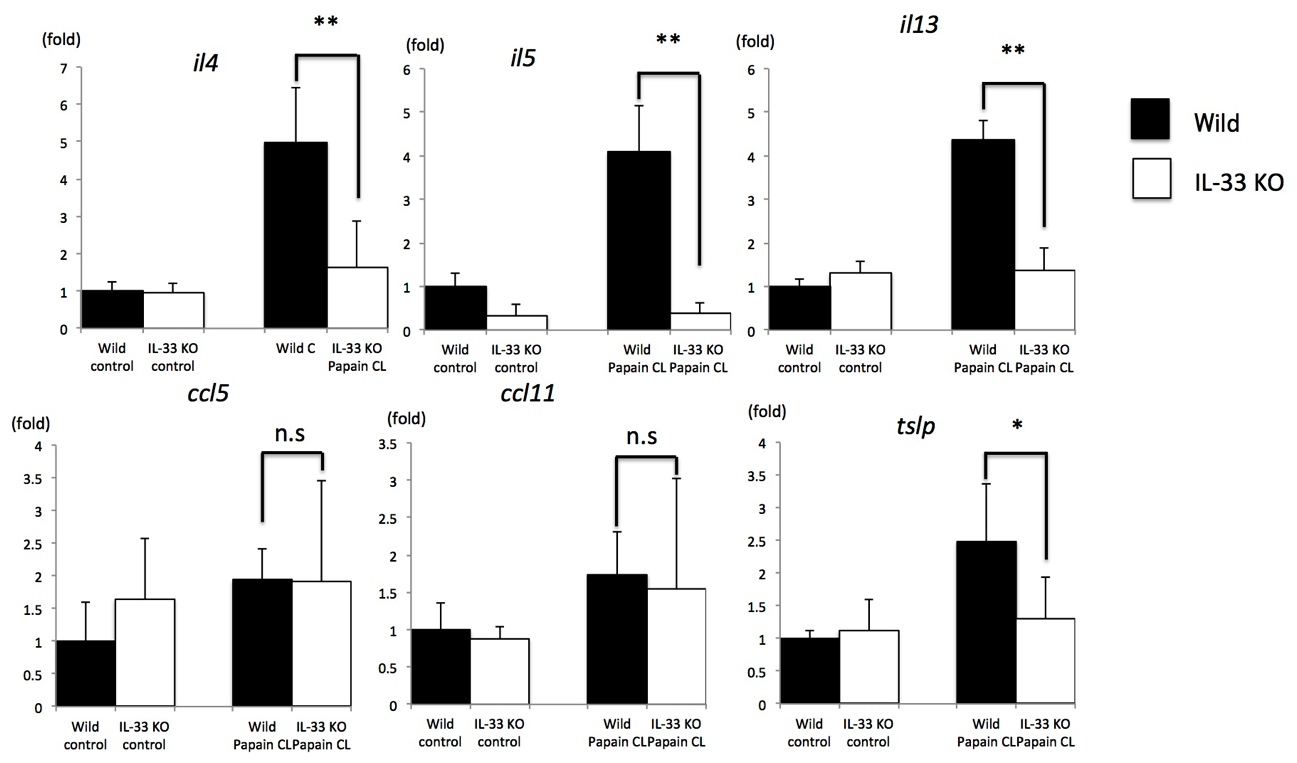
**

**Supplementary Figure 7: Cytokine expression in papain-CL model using TSLPR KO mice**

**
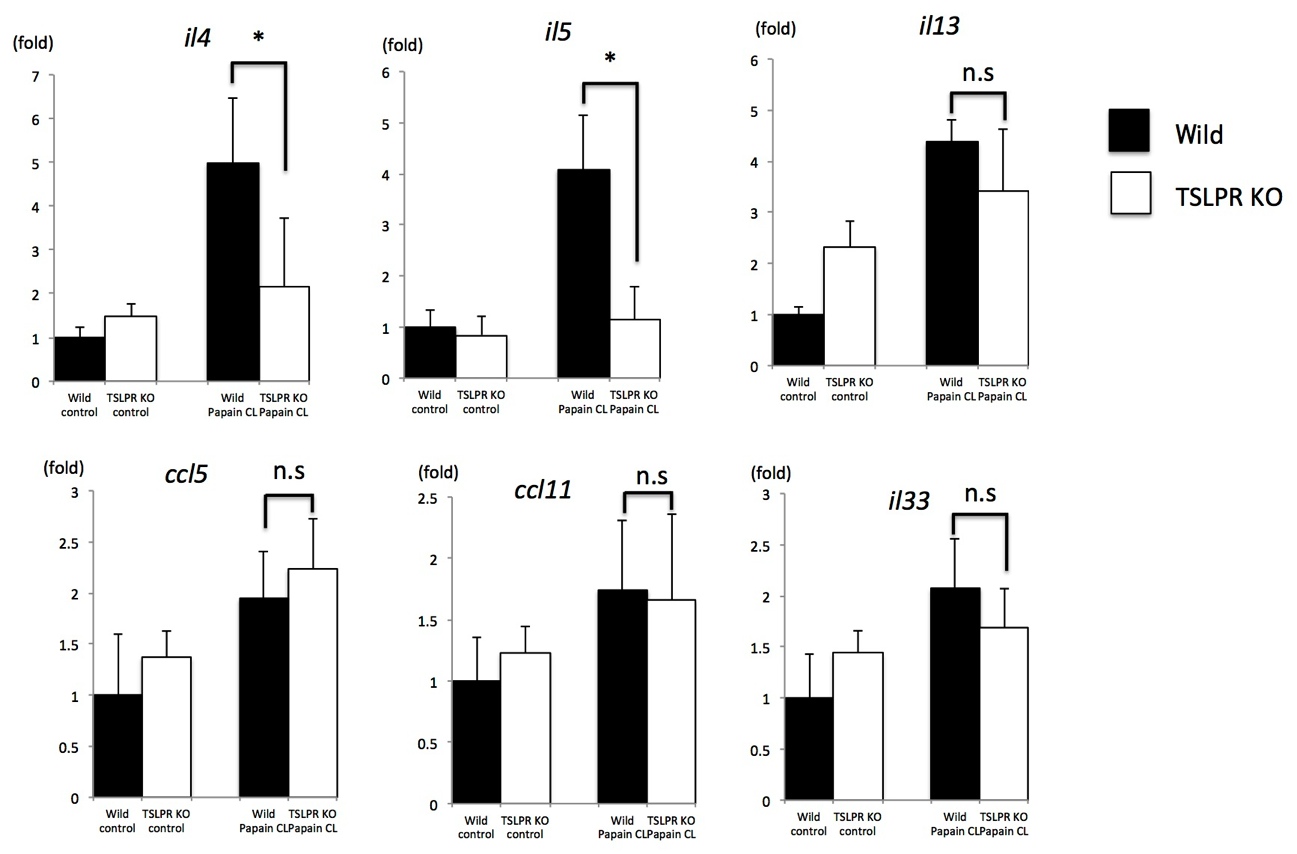
**

**Supplementary Figure 8: Diphtheria toxin (DT) treatment of Bas-TREK mice depleted basophils in papain-CL models**

**
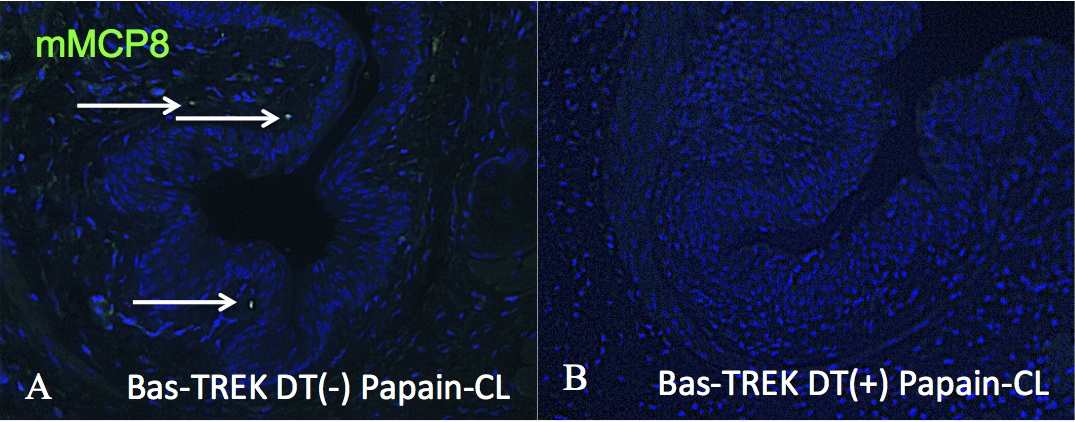
**

**Supplementary Figure 9: Diphtheria toxin (DT) treatment of Bas-TREK mice reduced Th2 cytokine expression in the conjunctiva of the papain-CL model**

**
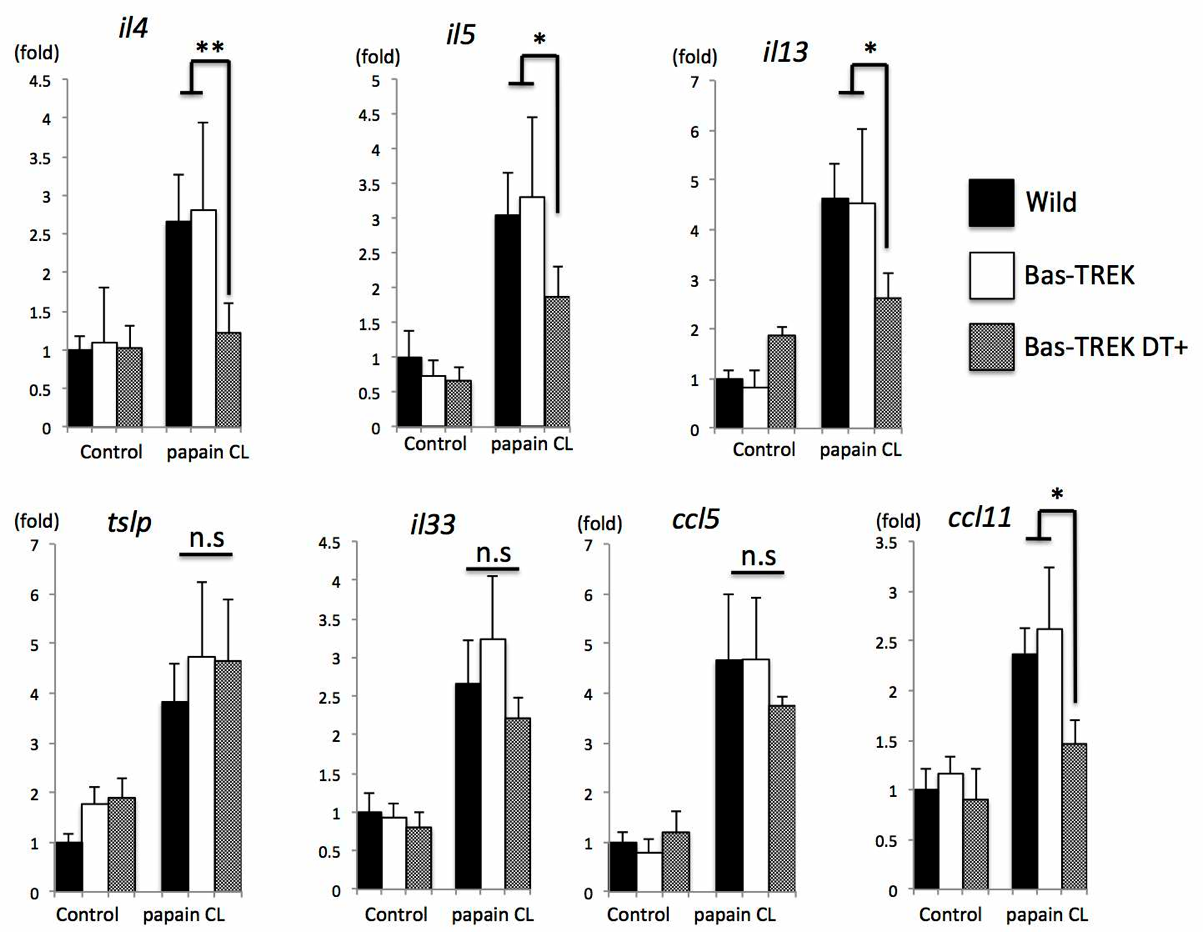
**

**Supplementary Figure 10: Papain-CL models using IL-4-deficient G4 mice**

**
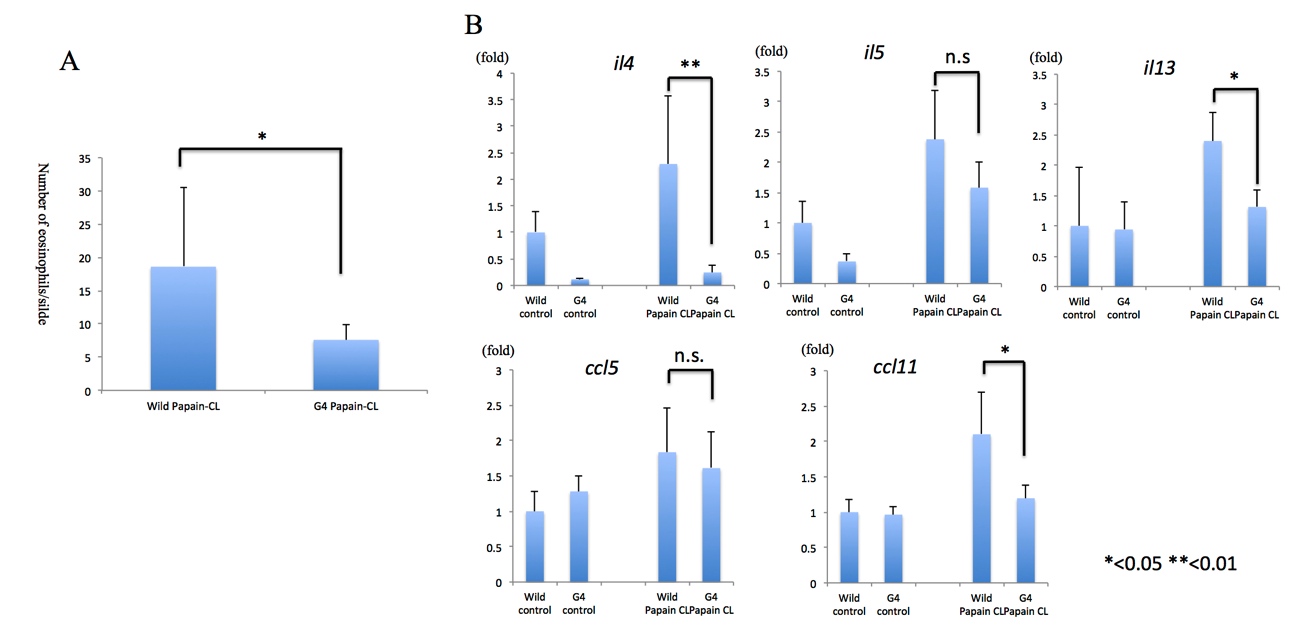
**

**Supplementary Figure 11: Depletion of ILC2 cells in the conjunctiva of Rag2 KO mice via intraperitoneal injection of an anti-CD25 antibody**

**
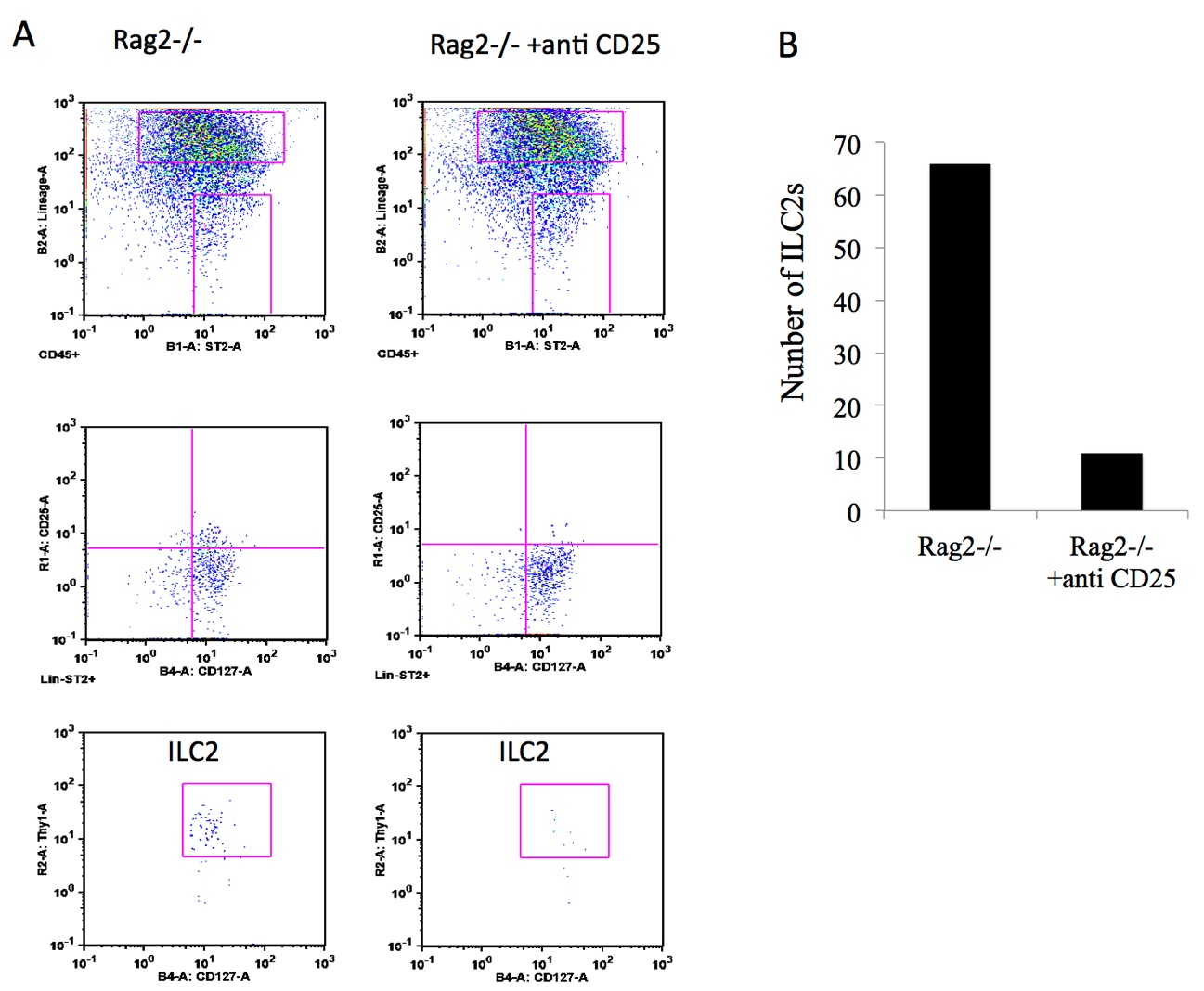
**

**Figure Legend for Supplementary Figure 1**

To evaluate the effect of the electrical charges of soft contact lenses (CLs) on the ability to retain papain, CLs (both negatively charged and positively charged) were incubated with papain solution for 24 hours. Then the papain-CLs were incubated with SDS sample buffer. Immunoblot analysis with an anti-papain Ab showed that papain was retained on negatively-charged CLs (left lane) even after PBS washing (middle lane), but not on positively-charged CLs (right lane).

**Methods for Figure 1**

**SDS-polyacrylamide gel electrophoresis (PAGE) and Western blotting**

Negatively-charged CLs 2mm in diameter were prepared by cutting out the centers of commercially available CLs (One day Acuve, Johnson and Johnson Japan, Tokyo, Japan) using a 2mm disposable trepan (Kai Industries, Tokyo, Japan) and then they were soaked with 25mg/ml papain (Wako, Tokyo, Japan) in phosphate-buffered saline (PBS) for 24 hours (papain-CL). As a negative control positively-charged CLs were obtained from SEED Co., LTD. (Tokyo, Japan). Papain-CLs were reacted with SDS-sample buffer (62.5mM Tris-HCl, pH 6.8, 2% SDS, 20% glycerol, and 0.04% bromophenol blue) for 1 hour at room temperature. Then 50 mM dithiothreitol (DTT) was added to the samples and they were further incubated for 15 minutes at 65℃. A 15µl alquot of each sample was loaded on 12% tris-glycine gel with Precision Blue prestained protein standards (Bio-Rad Japan, Tokyo, Japan). The electrophoresed protein was transferred to a polyvinylidene fluoride (PVDF) membrane (Pall Japan, Tokyo, Japan) that was then incubated with affinity purified rabbit anti-papain antibody (obtained from Bethyl Laboratories Inc. Montgomery, TX, used in 1:1000 dilution in Block-One blocking solution, Nacalai Tesque, Kyoto, Japan) overnight at 4℃. After washing with Tris-buffered saline (10mM Tris-HCl, pH 7.6, 150mM NaCl) containing 0.05% Tween 20 (TBS-T), the membrane was incubated with a 1:10,000 dilution of HRP-conjugated anti-rabbit IgG. (GE Healthcare, Uppsala, Sweden) for 1 hour and then visualized with ECL Plus Western blotting reagents (GE Healthcare).

**Figure legend for Supplementary Figure 2.** Papain-contact lens (papain-CL)-induced inflammation in the mouse eye. On day 0, a papain-CL was inserted into the conjunctival-sac of the right eye (A), and the eyelid was sutured with 8-0 nylon (B). The papain-CL was exchanged once on day 2, and the second papain-CL was removed and the eye was sampled for further analysis on day 5 (C).

**Figure legend for Supplementary Figure 3**

We counted the numbers of eosinophil in the conjuctival epithelium and substantia propria, except within the lumina of vessels, through the corneal limbus to the mucocutaneous junction via the conjunctival fornix.

**Figure legend for Supplementary Figure 4**

Giemsa staining showed comparable numbers of eosinophils (arrows) in wild-type mice (A) and Rag2 KO mice (B), but significantly fewer infiltrating eosinophils were observed in wild-type mice wearing heat-inactivated papain-CLs (C). Numbers of eosinophils in the conjunctivae per slide are shown (D). N.S.: No significant difference. Error bars show means ± SD (**P<0.05*, n=5 per group) Representative data from two independent experiments are shown.

**Figure Legend for Supplementary Figure 5**

Total serum IgE levels on day 0 (before administration) and day 5 (after final papain-CL removal) determined using ELISA MAX mouse IgE ELISA kits (Biolegend, San Diego, CA). When mice in the same genotype group were compered, there was no significant upregulation of the total IgE concentration after papain-CL administration. The data are representative of triplicate measurements. Error bars show means ± SD (**P<0.05*, n=3 per group). Representative data from three independent experiments are shown.

**Figure legend for Supplementary Figure 6**

Cytokine/chemokine (*il4, il5, il13*, *ccl5, ccl11,* *tslp*) mRNA expression in the conjunctivae of papain-CL models using wild-type and IL-33 KO mice was quantified by real-time PCR. Relative mRNA expression is shown as fold changes of mRNA expression levels in contralateral conjunctival tissue (control) of the wild-type mice. Data representative of triplicate measurements of three mice per group, carried out as three independent experiments, are shown. (**P<0.05, **P<0.01*)

**Figure legend for Supplementary Figure 7**

Cytokine/chemokine (*il4, il5, il13*, *ccl5, ccl11,* *il33*) mRNA expression in the conjunctivae of papain-CL conjunctivitis models using wild-type and TSLPR KO mice was quantified by real-time PCR. Relative mRNA expression is shown as fold changes of mRNA expression levels in contralateral conjunctival tissue (control) of the wild-type mice. Data representative of triplicate measurements of three mice per group, carried out as three independent experiments, are shown. (**P<0.05*).

**Figure Legend for Supplementary Figure 8**

mMCP8 immunostaining was carried out to confirm the efficiency of diphtheria toxin (DT) treatment-induced basophil depletion in Bas-TRECK mice. No basophils were observed in the papain-CL model with DT treatment (B). On the other hand, basophils were detected in the papain-CL model without DT treatment (A). Representative data from two independent experiments are shown. Original magnification x200.

**Figure Legend for Supplementary Figure 9**

The expression of cytokines/chemokines (*il4, il5, il13*, *tslp, il33, ccl5,* and *ccl11*) in papain-CL model was compared among wild-type, Bas-TRECK mice without DT treatment, and DT-treated Bas-TRECK mice. Significant reduction of *il4, il5, il13 and ccl11* mRNA expression was observed in papain-CL model using Bas-TRECK mice. (**P*<0.05 and ***P*<0.01, n=3 per group). Representative data from two independent experiments are shown.

**Figure Legend for Supplementary Figure 10**

Reduction of infiltrating eosinophil numbers (A) was observed in the papain-CL model in IL-4 KO (G4) mice (**P*<0.05, n=5 per group). Expression of inflammatory cytokine/chemokine (*il4, il5, il13*, *ccl5,* and *ccl11*) mRNAs was quantified by real-time PCR. Significantly attenuated expression of *il4 and il13* mRNAs was observed in the papain-CL model IL-4 KO mice compared to the wild-type mice. No differential *il5* and *ccl5* mRNA expression was observed. Data representative of triplicate measurements, carried out as two independent experiments, are shown. (**P*<0.05, n=3 per group)

**Figure Legend for Supplementary Figure 11**

Conjunctival tissues isolated from 10 eyes of isotype-matched mAb-treated Rag2 KO mice and from 10 eyes of anti-CD25 mAb-treated Rag2 KO mice were analyzed by FACS (A). Total numbers of ILC2s detected in the conjunctival tissues were counted (B). Representative data from two independent experiments are shown.


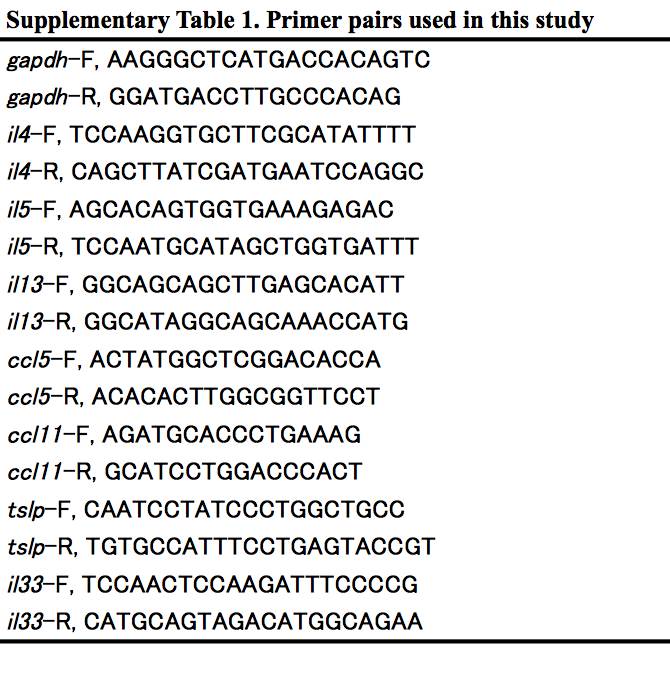

Supplement: Supplementary file 1 — Figure S1. To evaluate the effect of the electrical charges of soft contact lenses (CLs) on the ability to retain papain, CLs (both negatively charged and positively charged) were incubated with papain solution for 24 h. Then the papain‐CLs were incubated with SDS sample buffer. Immunoblot analysis with an anti‐papain Ab showed that papain was retained on negatively‐charged CLs (left lane) even after PBS washing (middle lane), but not on positively‐charged CLs (right lane). Figure S2. Papain‐contact lens (papain‐CL)‐induced inflammation in the mouse eye. On day 0, a papain‐CL was inserted into the conjunctival‐sac of the right eye (A), and the eyelid was sutured with 8‐0 nylon (B). The papain‐CL was exchanged once on day 2, and the second papain‐CL was removed and the eye was sampled for further analysis on day 5 (C). Figure S3. We counted the numbers of eosinophil in the conjuctival epithelium and substantia propria, except within the lumina of vessels, through the corneal limbus to the mucocutaneous junction via the conjunctival fornix. Figure S4. Giemsa staining showed comparable numbers of eosinophils (arrows) in wild‐type mice (A) and Rag2 KO mice (B), but significantly fewer infiltrating eosinophils were observed in wild‐type mice wearing heat‐inactivated papain‐CLs (C). Numbers of eosinophils in the conjunctivae per slide are shown (D). N.S.: No significant difference. Error bars show means ± SD (*P < 0.05, n = 5 per group) Representative data from two independent experiments are shown. Figure S5. Total serum IgE levels on day 0 (before administration) and day 5 (after final papain‐CL removal) determined using ELISA MAX mouse IgE ELISA kits (Biolegend, San Diego, CA). When mice in the same genotype group were compered, there was no significant upregulation of the total IgE concentration after papain‐CL administration. The data are representative of triplicate measurements. Error bars show means ± SD (*P < 0.05, n = 3 per group). Representative data from three [file IID3-5-515-s001.doc]
